# Investigating the relationship between aging perception and self-efficacy in the older adults: a cross-sectional study in Eastern Iran

**DOI:** 10.1186/s12877-024-05231-7

**Published:** 2024-08-01

**Authors:** Mohsen Arjmand-sangani, Gholamreza Sharifzadeh, Narges Soltani, Marzieh Torshizi

**Affiliations:** 1 Department of Nursing, School of Nursing and Midwifery, Birjand University of Medical Sciences, Birjand, Iran; 2 Department of Epidemiology and Biostatistics, School of Health, Social Determinants of Health Research Center, Birjand University of Medical Sciences, Birjand, Iran; 3 Instructor of Midwifery, Department of Midwifery, School of Nursing and Midwifery, Birjand University of Medical Sciences, Birjand, Iran

**Keywords:** Perception of aging, Self-efficacy, Cross-sectional studies, Aged

## Abstract

**Background:**

With the global increase in the older adults population, understanding factors that impact their quality of life is crucial. The perception of aging and self-efficacy are significant factors affecting older adults health outcomes. This descriptive cross-sectional study investigates the relationship between Aging Perception and self-efficacyamong older adults individuals in Birjand city, a demographic that is rapidly increasing but under-research.

**Methods:**

This cross-sectional study was conducted from September 2022 to September 2023 on 400 older adults individuals in Birjand city. Participants were selected using a random sampling method from four regions, ensuring a representative sample. Data were collected through two main questionnaires: the Shortened Perceived Aging Questionnaire (SPAQ) and the General Self-Efficacy Questionnaire (GSE), both validated for the Persian-speaking population. Statistical analysis was performed using SPSS SOFTWARE. ARMONK, NY: IBM CORP. version 26, employing nonparametric tests due to the non-normal distribution of data.

**Results:**

The study found a strong positive correlation between Aging Perception and self-efficacy (Spearman’s *R* = 0.79, *p* < 0.001), indicating that a more positive perception of aging is associated with higher self-efficacy. The analysis also revealed that men generally reported a more positive perception of aging and higher self-efficacy compared to women. However, underlying diseases and marital status did not significantly affect the Aging Perception or self-efficacy scores.

**Conclusions:**

The findings suggest that enhancing self-efficacy among the older adults could improve their perception of aging, potentially leading to better health outcomes and quality of life. This study underscores the need for targeted interventions that consider cultural and gender-specific factors. Further research using longitudinal designs is recommended to explore the causality between Aging Perception and self-efficacy and to confirm these findings across different regions and cultural backgrounds in Iran.

**Supplementary Information:**

The online version contains supplementary material available at 10.1186/s12877-024-05231-7.

## Introduction

According to the World Health Organization (WHO), the number of older adults worldwide is rapidly increasing. In 2023, there were an estimated 961 million people aged 60 years and older, and this number is projected to reach 2 billion by 2025. Notably, the majority of this growing population will reside in developing countries [1, 2]. In Iran, for instance, the 2016 census revealed that 3.9% of the population was over 60 years old, and this figure is expected to rise to 31.5% by 2050 [3]. In comparison to other West Asia and Middle-Eastern region countries, Iran’s aging population is growing at a similar rate, with countries such as Turkey and Egypt also experiencing significant increases in their older adults populations [4, 5].

Aging is a natural and inevitable part of life, characterized by physiological and psychological changes. It is a complex process that affects all living beings, including humans [6].

The perception of aging refers to an individual’s subjective view of their own aging process and their level of satisfaction with it. This perception reflects how well the individual has adapted to the various changes associated with aging. Importantly, the perception of aging has a significant impact on an older adult’s behavior and relationships with others. A negative perception of aging can lead to a decline in overall functioning [3].

Research has established a clear connection between the perception of aging and various health and psychological variables, including life satisfaction, depression, anxiety, daily living activities, and physical activity in older adults [7].

One such variable is self-efficacy, which refers to an individual’s confidence in their ability to achieve specific goals. Individuals with higher self-efficacy tend to enjoy better overall health [8, 9, 10].

Studies have shown that low self-efficacy is associated with various psychological and emotional disorders, as well as behavioral dysfunctions in older adults [9, 11–17].

Self-efficacy directly and indirectly influences individuals’ health behaviors and plays a crucial role in achieving goals, performing tasks, and coping with various challenges [18].

Self-efficacy expectations in specific domains may be influenced by stereotypes associated with aging, which can be reinforced through both positive and negative personal experiences.

Furthermore, perceived self-efficacy serves as a powerful source of control over behavior and plays a significant role in psychological adjustment, maintaining positive mental and physical health, and overall well-being [19, 20].

Several studies have explored the relationship between perception of aging and self-efficacy. For instance, a study by Havighurst et al. (2002) found that perception of aging significantly influences coping patterns (*p* < 0.001) [19]. Additionally, a study by Jang et al. (2015) revealed that older adults with lower education levels, lower socioeconomic status, and multiple health problems had a more negative perception of aging and health [21].

Given that the perception of aging can vary across societies and over time, researchers have emphasized the need for further research in this field. This is particularly relevant in the context of Iran, where the population of older adults is rapidly growing [3, 7, 22].

Considering the importance of empowering and improving the quality of life for older adults, and the lack of research on the relationship between perception of aging and self-efficacy in the Iranian context, this study aims to investigate this relationship among older adults in Iran.

## Methods

### Setting and study design

The study was a cross-sectional descriptive study conducted on 400 older adults people in Birjand city from September 2022 to September 2023.

### Populations, inclusion, and exclusion criteria

This study included older adults in Birjand city who were willing to participate, aged 60 years or older, had no history of neurological or psychiatric disorders, and did not have sensory impairments such as blindness or deafness. Participants were excluded if they were unwilling to participate in the study or if they did not complete the questionnaires.

### Measurement instruments

The following questionnaires were used to collect data:

Demographic Characteristics Questionnaire: This questionnaire collected information about the participants’ age, gender, marital status, number of children, and underlying diseases.

Shortened Perceived Aging Questionnaire (SPAQ): This 17-item questionnaire measures an individual’s perception and feelings about aging. The dimensions of Perceived Aging included progressive course, positive results, positive control, results and negative control, and emotional reactions. The questionnaire is scored on a 5-point Likert scale from 1 (strongly disagree) to 5 (strongly agree). The scoring for the control and negative outcomes subscales is reversed. The lowest possible score is 17 and the highest is 85, with a higher score indicating a deeper understanding of aging.

The English version of the questionnaire was developed by Barker et al. (2007) in Ireland [23] and is provided as a supplementary file. The questionnaire has been translated and validated into Persian by Sadeghi Moghadam et al. (2016) [24]. The Cronbach’s alpha coefficient for the dimensions and the total questionnaire was 0.75, and the test-retest reliability coefficient was 0.94, indicating good validity and reliability of the Persian version of the questionnaire.

To determine the content validity of the SPAQ in the present study, it was given to 10 faculty members of the Birjand University of Nursing and Midwifery. After making the necessary modifications, the reliability of the instruments was assessed using Cronbach’s alpha coefficient. The questionnaires were administered to 20 eligible research units, and the Cronbach’s alpha coefficient was calculated as 0.90. The SPAQ was completed by the older adults once, on the day of the interview.

General Self-Efficacy Questionnaire (GSE): This 17-item questionnaire, developed by Sherer and Maddux (1982) [25], measures an individual’s general self-efficacy. It is scored on a 5-point Likert scale from 1 (strongly disagree) to 5 (strongly agree). Items 1, 3, 8, 9, 13, and 15 are scored in reverse. The minimum score is 17 and the maximum is 85.

The English version of the questionnaire is provided as a supplementary file. Sherer and Maddux reported the validity of the GSE as 0.86, and Woodruff and Cashman (1982) [5] reported its validity as 0.84. Ali Nikaroui (2015) [26] obtained a Cronbach’s alpha coefficient of 0.78 for the scale. Ganji and Farajiani (2016) [27] reported the reliability of the instrument using Cronbach’s alpha coefficient as 0.81, and its content validity was also reported as good.

To determine the content validity of the GSE in the present study, it was given to 10 faculty members of the Birjand University of Nursing and Midwifery. After making the necessary modifications, the reliability of the instruments was assessed using Cronbach’s alpha coefficient. The questionnaires were administered to 20 eligible research units, and the Cronbach’s alpha coefficient was calculated as 0.82. The GSE was completed by the older adults once, on the day of the interview.

### Sampling

The study was conducted in Birjand city, Iran, from September 2022 to September 2023. In the first stage, the city was divided into four regions, and one health center was randomly selected from each region.

In the next stage, the researcher visited each health center in person and extracted the list of older adults people covered by that center from the SIB (Integrated Health System) system. The SIB system is the latest electronic health record system designed in Iran. It was officially launched in February 2016 with the aim of integrating health information and providing health and treatment services as part of the health sector programs in the Health Transformation Plan throughout the country.

Then, 100 older adults people were randomly selected from the list extracted from the SIB system. If they met the inclusion criteria, the center’s liaison contacted the older adults person and, after explaining the objectives of the research, invited them to participate in the study. A quiet room was chosen in the center for the interview with the older adults person.

If the older adults person was not willing to come to the health center, the interview was conducted at their home with their prior consent. If the older adults person was not willing to cooperate, another person was selected from the list.

The interview time was selected based on the convenience and suggestion of the older adults person. For older adults people who were unable to read and write, each questionnaire was read to them and each item was filled out based on their opinion and choice.

### Sample size

The sample size was calculated using a power analysis to ensure adequate representation of the older adults population in Eastern Iran. Based on a previous study [3], we estimated a moderate effect size of 0.5 for the relationship between aging perception and self-efficacy. Using a power analysis with an alpha level of 0.05 and a power of 0.8, we determined that a minimum sample size of 384 participants was required. To account for potential non-response and incomplete questionnaires, we added 16 participants to the sample, resulting in a final sample size of 400 participants.

### Statistical analysis

After data collection and ensuring the accuracy of data entry, descriptive statistics (frequency percentage, mean, standard deviation) were analyzed using SPSS SOFTWARE. ARMONK, NY: IBM CORP. version 26 software in the descriptive statistics section.

In the inferential statistics section, the normality of quantitative variables was first checked using the Kolmogorov-Smirnov test. Since the data did not have a normal distribution, nonparametric analytical tests (Spearman correlation coefficient, Mann-Whitney U test, and Kruskal-Wallis test) were used to analyze the data. A significance level of α = 0.05 was considered.

## Results

The study involved 400 older adults in Birjand city, Iran, with a mean age of 75.4 ± 9.7 years, ranging from 60 to 92 years. The participants had a mean of 3.1 ± 2.1 children, with a minimum of 0 and a maximum of 8 children (Table 1).

**Table 1:** Demographic characteristics of the participants

Variable	
Age (Mean ± SD)	75.40 ± 9.70
Number of children (Mean ± SD)	3.10 ± 2.10
Sex n(%)	
Female	223 (55.70)
Male	177 (44.30)
Marital status n(%)	
Single	56 (14.00)
Married	212 (53.00)
deceased wife	96 (24.00)
Divorced	36 (9.00)
Underlying disease n(%)	
Yes	238 (59.50)
No	162 (40.50)
Living with n(%)	
Alone	76 (19.00)
With wife	137 (34.00)
With children	97 (24.50)
With wife and children	87 (22.00)
With other	3 (0.50)

The analysis of the data revealed that the mean self-efficacy score was 40.5, with a standard deviation of 12.1, and scores ranging from 21 to 65. The total score of perception of aging had a mean of 51.1, with a standard deviation of 8.7, and scores ranging from 38 to 69. The dimensions of perception of aging, including progressive course, positive results, positive control, results and negative control, and emotional reactions, had mean scores ranging from 8.1 to 13.3, with varying standard deviations (Table 2).

**Table 2:** Mean and standard deviation of self-efficacy score and perception of aging and dimensions of perception of aging

Variable		Frequency	Minimum	Maximum	Mean ± SD
Efficacy		400	21	65	40.50 ± 12.10
Dimensions of Perception of Aging	Progressive course	400	3	15	8.10 ± 3.60
	Positive results	400	7	15	10.70 ± 2.40
	Positive control	400	6	15	12.00 ± 12.60
	Results and negative control	400	5	25	13.30 ± 5.00
	emotional reactions	400	3	15	7.00 ± 3.10
Total score of perception of aging		400	38	69	51.10 ± 8.70

A strong positive correlation was observed between perception of aging and self-efficacy, with a Spearman correlation coefficient of *R* = 0.79 and a p-value < 0.001, indicating a statistically significant relationship (Table 3).

**Table 3:** The relationship between perception of aging and self-efficacy

Variable	*P*
relationship between perception of aging and self-efficacy	*R* = 0.79 *P* < 0.001
spearman correlation	

The comparison of the average score of perception of aging based on demographic variables showed that male participants had a significantly higher mean score than females (52.5 ± 8.8 vs. 49.9 ± 8.4, *p* = 0.003), as determined by the Mann-Whitney U test. However, marital status did not significantly affect the mean scores (*p* = 0.55), according to the Kruskal-Wallis test. The presence of an underlying disease did not have a statistically significant effect on the mean perception of aging scores (*p* = 0.131), with participants with underlying diseases scoring slightly lower (50.4 ± 8.3) than those without (52.0 ± 9.0) (Table 4).

**Table 4:** Comparison of the average score of perception of aging based on demographic variables

Variable	Mean ± SD	*P*
Sex		
Female	49.90 ± 8.40	*P = 0.003
Male	52.50 ± 8.80	
Marital status		
Single	50.00 ± 8.80	**P = 0.55
Married	51.50 ± 9.00	
Deceased wife	51.10 ± 8.30	
Divorced	49.90 ± 7.30	
Underlying disease		
Yes	50.40 ± 8.30	*P = 0.13
No	52.00 ± 9.00	
Level of education		
Illiterate	50.60 ± 8.10	**P = 0.26
Below diploma	50.20 ± 8.60	
Diploma	51.50 ± 9.20	
Associate	52.5 ± 9.10	
Bachelor and above	54.70 ± 9.60	

* Mann–Whitney U test **Kruskal-wallis

The analysis of self-efficacy scores based on demographic variables indicated a marginally significant difference between female and male participants (39.5 ± 11.7 vs. 41.8 ± 12.3, *p* = 0.052). Marital status did not significantly affect the mean self-efficacy scores (*p* = 0.48), and the difference in scores between those with and without underlying diseases was not statistically significant (*p* = 0.37) (Table 5).

**Table 5:** Comparison of the average score of self-efficacy based on demographic variables

Variable	Mean ± SD	*P*
Sex		
Female	39.50 ± 11.70	*P = 0.052
Male	41.80 ± 12.30	
Marital status		
Single	39.40 ± 11.80	**P = 0.48
Married	41.20 ± 12.00	
Deceased wife	40.20 ± 12.60	
Divorced	38.50 ± 11.00	
Underlying disease		
Yes	39.40 ± 11.60	*P = 0.037
No	42.00 ± 12.50	
Level of education		
Illiterate	39.70 ± 11.70	**P = 0.070
Below diploma	39.30 ± 11.90	
Diploma	41.50 ± 12.20	
Associate	41.10 ± 12.70	
Bachelor and above	47.20 ± 10.80	

* Mann–Whitney U test **Kruskal-wallis

## Discussion

The present study aimed to investigate the relationship between perceived aging and self-efficacy in older adults referring to comprehensive health service centers in Birjand city, Iran. Several studies have been conducted in different societies on the perceived aging and its effect on various aspects of older adults’ lives. In some of these studies, the level of perceived aging was reported to be low, while in others it was reported to be higher. The reason for these differences may be due to cultural differences and social factors that probably affect individual experiences of aging [28–31].

The mean score of perceived aging in our study was 51.1, with a standard deviation of 8.7, ranging from a minimum of 38 to a maximum of 69. These figures suggest a moderate level of perceived aging among the older adults in Birjand city, Iran.

According to the results of the present study, the mean score of perceived aging in men (52.5) was higher than that in women (49.9), which was statistically significant. This finding is consistent with the study of Mir Emadi et al. [32], who showed that perceived aging was significantly higher in older adults men than in older adults women. The study of Minhat et al. [1] in middle-aged women in Malaysia showed that they thought seriously about the possible negative experiences associated with aging and being old, but few of them experienced anxiety about aging. The occurrence of anxiety about aging was related to their personal experiences and observations of the surrounding society [30]. On the other hand, research has shown that women have a more negative perception of their own aging than men, and this issue is directly related to the common cultural stereotypes in each society, especially when emphasis is placed on physical appearance and biological changes associated with increasing age [33].

In the present study, a positive and significant correlation was observed between the perceived aging score and self-efficacy (*p* < 0.001), which is consistent with the study of Hava Tavoli et al. [19] on older adults over 75 years old, which showed that perceived aging has an effect on self-efficacy. The results of the present study showed that the mean score of self-efficacy in older adults in Birjand city was 40.5 ± 12.1, which was moderate. In the study of Salhi et al. [29], the mean score of self-efficacy in older adults living in Kahrizak nursing home was also evaluated as moderate. In the study of Azimiyan et al. [34], the majority of older adults in Ramsar city had moderate self-efficacy. Uchenwoke et al. [35] also evaluated the self-efficacy of people with mobility disabilities using assistive devices in selected communities in Nigeria as moderate. All of the above cases support the results obtained in the present study.

While the findings of the study by Kahe et al. [28] among older adults living in nursing homes under the supervision of the Welfare Organization of Tehran showed that the mean self-efficacy in these people was low, the findings of the study by Kashani Nia et al. [36] on older adults in Jiroft city showed that the majority of the older adults studied had high self-efficacy. In the research of Jamal-Livani et al. [37], the self-efficacy of older adults referring to Imam Khomeini Hospital in Tehran was also evaluated as above average.

The findings of the present study showed that the mean score of self-efficacy in older adults without underlying diseases was higher than that in older adults with underlying diseases, which was statistically significant. This finding was consistent with the results of the studies by Emamghlizadeh Baboli et al. [38] and Aslani et al. [39]. In the study of Todorova et al. [40], which was conducted among 179 adults and older adults with physical disabilities in Stara Zagora, Bulgaria, the same result was also reported. In fact, having underlying diseases increases individual problems such as pain, fatigue, and hopelessness, and involvement with more problems and reduced social interactions and participation, all of which are very effective in reducing self-efficacy.

According to the results of the present study, the marital status of older adults was not significantly correlated with their self-efficacy, which was consistent with the study of Torki et al. [41] who evaluated the self-efficacy of residents of nursing homes in the west of Tehran, and the study of Kashani Nia et al. [36].

The present study found that the mean self-efficacy score of men was higher than that of women, although this difference was not statistically significant. This finding is inconsistent with the study of Tore Bonsaksen et al. [42] in the Norwegian population, which showed that men had higher self-efficacy than women. However, the results of the present study are consistent with the studies of Azimiyan et al. [34], Doba et al. [43], and Cybulski et al. [44] which did not find a significant relationship between gender and self-efficacy in older adults.

To promote self-efficacy and perceived aging, it is essential to consider the cultural and social context of Iran. The study revealed that men generally reported higher self-efficacy and a more positive perception of aging compared to women, indicating the need for gender-specific interventions. These interventions could include educational programs aimed at improving self-efficacy and challenging negative perceptions of aging, particularly among older women.

Furthermore, while the presence of underlying diseases did not significantly affect the perception of aging or self-efficacy scores, it is important to address the health concerns of the older adults to ensure their physical and mental well-being. Health promotion programs that focus on disease management and prevention could indirectly support the enhancement of self-efficacy and perceived aging by improving the overall health status of the older adults.

Community-based initiatives that encourage social participation and engagement can also play a vital role in promoting self-efficacy and a positive perception of aging. By providing opportunities for older adults to contribute to their communities and maintain social connections, these initiatives can help combat feelings of isolation and increase feelings of competence and self-worth.

### Strengths

The current study has several strengths. Firstly, it utilizes a large sample size from the older adults population in Birjand City, Iran, which enhances the reliability of the results. Secondly, it provides a comprehensive and balanced review of the literature, lending credibility to the research. The study also employs highly accurate measurement tools for data collection, ensuring the precision of the findings. The utilization of both descriptive and inferential statistics for data analysis allows for a thorough examination of the data. The article is well-organized and written in a clear and concise manner. Lastly, this study contributes to a better understanding of the relationship between aging perception and self-efficacy in the Iranian older adults population, which could be beneficial for future research and policy-making.

### Limitations

Despite its strengths, the study does have some limitations. The cross-sectional design means it can’t determine causality, only correlations. The sampling method, which involved selecting participants from comprehensive health service centers, may introduce selection bias as these individuals might not represent the overall older adults population. The use of self-report questionnaires can also lead to self-reporting bias, which could potentially skew the results. The findings may not be generalizable to other older adults populations in Iran or other countries because of cultural and social differences. Finally, the study didn’t include individuals with neurological or psychiatric disorders or sensory impairments such as blindness or deafness, thereby limiting the scope of the research.

## Conclusion

This study found a significant positive correlation between perceived aging and self-efficacy among older adults in Birjand City, Iran. Men reported higher self-efficacy and a more positive perception of aging compared to women, suggesting the need for gender-specific interventions. While underlying diseases and marital status did not significantly affect these scores, addressing health concerns is crucial for overall well-being.

These findings contribute to our understanding of the relationship between perceived aging and self-efficacy in the Iranian context. They highlight the importance of self-efficacy in shaping individuals’ experiences of aging and suggest that interventions targeting gender-specific factors could be beneficial. Future research with longitudinal designs and more diverse samples is needed to further explore the causal relationships and generalizability of these findings.

### Electronic supplementary material

Below is the link to the electronic supplementary material.

Supplementary Material 1

## Data Availability

The datasets generated in the current study are available from the corresponding author upon reasonable request.

## Abbreviations

WHO: World Health Organization
SPAQ: Shortened Perceived Aging Questionnaire
