# Supplementary material for: Investigating the relationship between aging perception and self-efficacy in the older adults: a cross-sectional study in Eastern Iran
Source: BMC Geriatr. 2024 Aug 1;24:649. doi: 10.1186/s12877-024-05231-7 (PMC11295669; doi:10.1186/s12877-024-05231-7)
Supplement: Supplementary file 1 — Supplementary Material 1 [file 12877_2024_5231_MOESM1_ESM.docx]

**supplementary file 1**

**Perceived Aging Questionnaire**

1. I always classify myself as old
2. I am always aware of the fact that I am getting older
3. I feel my age in everything that I do
4. As I get older I get wiser
5. As I get older I continue to grow as a person
6. As I get older I appreciate things more
7. I get depressed when I think about how ageing might affect the things that I can do
8. The quality of my social life in later years depends on me
9. The quality of my relationships with others in later life depends on me
10. Whether I continue living life to the full depends on me
11. Getting older makes me less independent
12. As I get older I can take part in fewer activities
13. As I get older I do not cope as well with problems that arise
14. Slowing down with age is not something I can control
15. I have no control over the effects which getting older has on my social life
16. I worry about the effects that getting older may have on my relationships with others
17. I feel angry when I think about getting older

**General Self-Efficacy Questionnaire**

1. When I make plans‚ I am certain I can make them work.
2. One of my problems is that I cannot get down to work when I should.
3. If I can’t do a job the first time I keep trying until I can.
4. When I set important goals for myself‚ I rarely achieve them.
5. I give up on things before completing them.
6. I avoid facing difficulties.
7. If something looks too complicated‚ I will not even bother to try it.
8. When I have something unpleasant to do‚ I stick to it until I finish it.
9. When I decide to do something new‚ I go right to work on it.
10. When trying to learn something new‚ I soon give up if I am not initially successful
11. When unexpected problems occur‚ I don’t handle them well.
12. I avoid trying to learn new things when they look too difficult for me.
13. Failure just makes me try harder.
14. I feel insecure about my ability to do things.
15. I am a self-reliant person.
16. I give up easily.
17. I do not seem capable of dealing with most problems that come up in life.
